# Radiation-induced liver disease after stereotactic body radiotherapy for small hepatocellular carcinoma: clinical and dose-volumetric parameters

**DOI:** 10.1186/1748-717X-8-249

**Published:** 2013-10-27

**Authors:** Jinhong Jung, Sang Min Yoon, So Yeon Kim, Byungchul Cho, Jin-hong Park, Su Ssan Kim, Si Yeol Song, Sang-wook Lee, Seung Do Ahn, Eun Kyung Choi, Jong Hoon Kim

**Affiliations:** 1 Department of Radiation Oncology, Asan Medical Center, University of Ulsan College of Medicine, 88, Olympic-ro 43-gil, Songpa-gu, Seoul 138-736, Republic of Korea; 2 Department of Radiology, Asan Medical Center, University of Ulsan College of Medicine, 88, Olympic-ro 43-gil, Songpa-gu, Seoul 138-736, Republic of Korea

**Keywords:** Hepatocellular carcinoma, Stereotactic body radiotherapy, Radiation-induced liver disease

## Abstract

**Background:**

To investigate the clinical and dose–volumetric parameters that predict the risk of radiation-induced liver disease (RILD) for patients with small, unresectable hepatocellular carcinoma (HCC) treated with stereotactic body radiotherapy (SBRT).

**Methods:**

Between March 2007 and December 2009, 92 patients with HCC treated with SBRT were reviewed for RILD within 3 months of completing treatment. RILD was evaluated according to the Common Terminology Criteria for Adverse Events, version 3.0. A dose of 10–20 Gy (median, 15 Gy) per fraction was given over 3–4 consecutive days for a total dose of 30–60 Gy (median, 45 Gy). The following clinical and dose–volumetric parameters were examined: age, gender, Child-Pugh class, presence of hepatitis B virus, gross tumor volume, normal liver volume, radiation dose, fraction size, mean dose to the normal liver, and normal liver volumes receiving from < 5 Gy to < 60 Gy (in increments of 5 Gy).

**Results:**

Seventeen (18.5%) of the 92 patients developed grade 2 or worse RILD after SBRT (49 patients in grade 1, 11 in grade 2, and 6 in ≥ grade 3). On univariate analysis, Child-Pugh class was identified as a significant clinical parameter, while normal liver volume and normal liver volumes receiving from < 15 Gy to < 60 Gy were the significant dose–volumetric parameters. Upon multivariate analysis, only Child-Pugh class was a significant parameter for predicting grade 2 or worse RILD.

**Conclusions:**

The Child-Pugh B cirrhosis was found to have a significantly greater susceptibility to the development of grade 2 or worse RILD after SBRT in patients with small, unresectable HCC. Additional efforts aimed at testing other models to predict the risk of RILD in a large series of HCC patients treated with SBRT are needed.

## Background

Hepatocellular carcinoma (HCC) is one of the most common malignancies, ranking sixth worldwide [1]. Surgical resection and liver transplantation are the primary treatment modalities for HCC [2-4]. However, strict criteria limit the pool of eligible patients for both approaches. Radiofrequency ablation (RFA) can also be used with curative intent for small HCC which is unsuitable for surgery [5,6]. However, the proximity of the tumor to the main blood vessels, gall bladder, diaphragm, and liver surface presents major restrictions to use of RFA. Consequently, overall, only 30–40% of patients may derive benefit from the various radical curative treatments due to the numerous clinical conditions [7]. Therefore, in these clinical settings, alternative local therapeutic modalities are urgently needed.

With the development of three-dimensional conformal radiotherapy (3D-CRT), image-guided treatment and the resulting accumulation of knowledge on partial-volume liver tolerance, high-dose radiation could be delivered to focal liver volumes, thereby allowing radiotherapy as an alternative option for small intrahepatic tumors [8]. Several studies demonstrated that stereotactic body radiotherapy (SBRT) with 24–60 Gy administered in three to six fractions achieved high local control and overall survival rates of 65–100% and 48–82% at 1 year, respectively [9-13]. While many studies have demonstrated that variable factors are associated with radiation-induced liver disease (RILD) after conventional radiotherapy, only a few have examined RILD after SBRT and their results focused on clinical outcome and local control [8,12,14-17]. Recently, one study was aimed at identifying the parameters to predict hepatic toxicity and the deterioration of hepatic function [18]. However, additional studies are warranted to confirm the previous results.

Thus, the aim of the present study is to analyze the clinical and dose–volumetric parameters that predict the risk of RILD in patients with small, unresectable HCC treated with SBRT.

## Methods

### Patient selection

Patients who underwent SBRT for primary or recurrent HCC were registered and the database was retrospectively reviewed between March 2007 and December 2009. Eligibility criteria were as follows: (1) HCC not treatable by surgery or percutaneous ablative therapies; (2) HCC confined to the liver without extrahepatic metastases; (3) HCC < 6 cm in the longest diameter, and the presence of up to three lesions; (4) HCC with no evidence of major vascular invasion; (5) Child-Pugh A or B liver function; (6) adequate residual functional liver volume; (7) a sufficient distance (> 2 cm) of the tumor(s) from adjacent organs at risk, including duodenum, stomach, colon, and spinal cord; (8) an incomplete response after transarterial chemoembolization (TACE) or unsuitable for TACE by a physician’s decision; and (9) no history of previous external beam radiotherapy.

From the registered patients, those with data allowing analyses of clinical and dose–volumetric parameters predictive of the risk of RILD were selected. Further selection criteria were as follows: (1) availability of dosimetric parameters of SBRT and (2) follow-up times >3 months, with available biochemical profiles after SBRT. This study was approved by the Institutional Review Board of the Asan Medical Center, and informed consent in writing was obtained from each patient in the study.

### SBRT procedure

At least one week before computed tomography (CT) simulation, three fiducial markers (Standard Gold Soft Tissue Markers, CIVCO Medical Solutions, Kalona, IA) were inserted into the liver parenchyma around the tumors under ultrasonographic guidance in almost all patients except who had surgical clips or compact iodized oil from previous treatment. Pillows and vacuum molds were used for patient immobilization: 4-dimensional CT simulation was carried out (GE LightSpeed RT 16; GE Healthcare, Waukesha, WI) with free breathing. CT series were sorted according to respiratory phase using 4D imaging software (Advantage 4D version 4.2; GE Healthcare). Gross tumor volume (GTV) was delineated based on the visible gross tumor as seen on the CT images at end-expiratory phase; extension based on movement within the gating phase (30–70%) from the GTV was set as the internal target volume (ITV). Planning target volume (PTV) margin was 5 mm from the ITV. SBRT was planned using coplanar and/or non-coplanar 3D-CRT method with energies of 6 or 15 MV and delivered using a Varian-iX machine equipped with On-Board Imager (OBI) and Millennium 120 multileaf collimators (Varian Medical Systems). A dose of 10–20 Gy (median, 15 Gy) per fraction was given over 3–4 consecutive days to a total dose of 30–60 Gy (median, 45 Gy). The chosen isodose covering PTV was between 85–90%, which was normalized to the center of the PTV. Contouring and treatment planning were done by a 3D-radiotherapy planning system (Eclipse V8.0; Varian Medical Systems) using pencil beam convolution algorithm with heterogeneity correction with spatial resolution of 2.5 mm. Image guidance, including cone-beam CT and gated fluoroscopy, was performed prior to administrating each fraction of SBRT using the OBI.

### Evaluation of RILD

All patients were examined during SBRT to assess acute toxicity. After treatment, the patients were followed up every 1–3 months. Follow-up consisted of physical examinations, complete blood counts, biochemical profiles, tumor markers, and dynamic CT or MRI studies. RILD was defined as an elevation in transaminases or alkaline phosphatase of at least 2.5- to 5-fold and/or in bilirubin of at least 1.5- to 3-fold compared to either the upper normal limit or the pretreatment level, corresponding to grade 2 or higher hepatic toxicity according to the Common Terminology Criteria for Adverse Events (CTCAE) version 3.0, and/or non-malignant ascites in the absence of disease progression within 3 months after SBRT. This endpoint was modified from the classic and non-classic RILD proposed by the Quantitative Analyses of Normal Tissue Effects in the Clinic (QUANTEC) recommendation [19].

### Statistical analysis

The probabilities of cumulative survival and time to progression were calculated using the Kaplan-Meier method and the survival differences were analyzed by the log-rank test.

The clinical and dose–volumetric parameters analyzed were age, gender, Child-Pugh class, presence of hepatitis B virus (HBV) infection, Eastern Cooperative Oncology Group performance score (ECOG PS), GTV, PTV, normal liver volume, radiation dose, fraction size, mean dose to the normal liver, dose to 33% of the normal liver (D_33%_), dose to 50% of the normal liver (D_50%_), and the normal liver volumes receiving from < 5 Gy to < 60 Gy in increments of 5 Gy (respectively, rV_5Gy_ (reverse-V_5Gy_)_,_ rV_10Gy_, rV_15Gy_, rV_20Gy_, rV_25Gy_, rV_30Gy_, rV_35Gy_, rV_40Gy_, rV_45Gy_, rV_50Gy_, rV_55Gy_, and rV_60Gy_). Chi-square and Student’s *t*-test, for clinical parameters, and binary logistic regression, for dose–volumetric parameters, were performed for univariate analysis of an association with the risk of grade 2 or higher RILD. Multivariate analyses included only those variables with a p-value < 0.05 as determined in the univariate analysis. All statistical analyses were performed using the SPSS statistical package (version 12.0; SPSS Inc., Chicago, IL).

## Results

### Patient characteristics

**Figure 1:**
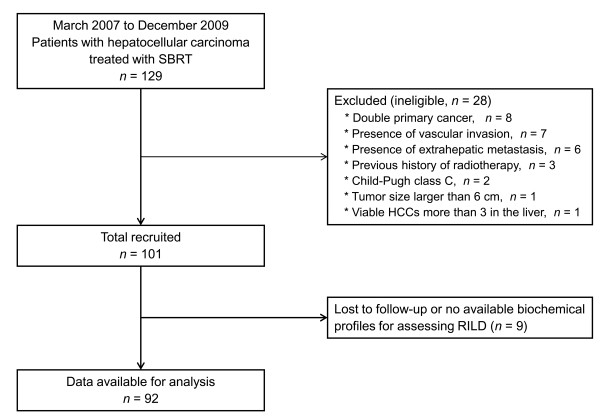
Flow diagram of the present study.

A total of 129 patients with HCC who were treated with SBRT were registered between March 2007 and December 2009 at our institution. Among these, 37 patients were excluded in the current analysis for the various reasons in Figure 1. The remaining 92 patients met the selection criteria. The characteristics and the dosimetric parameters of patients are summarized in Tables 1 and 2, respectively.

**Table 1:** Patient characteristics

**Variables**	**No. of patients (%)**
Gender	
Male	74 (80.4)
Female	18 (19.6)
Age (years)	
Median	61
Range	42–86
ECOG performance score	
0–1	89 (96.7)
2	3 (3.3)
Child-Pugh class	
A	68 (73.9)
B	24 (26.1)
Viral etiology	
HBs-Ag (+)	69 (75.0)
HBs-Ag (-)	23 (25.0)
Tumor volume (cm^3^)	
Median	8.6
Range	0.6–125.3
Previous treatments	
None	1 (1.1)
TACE	47 (51.1)
TACE, RFA	21 (22.8)
TACE, PEIT	4 (4.3)
TACE, RFA, PEIT	2 (2.2)
Resection	1 (1.1)
Resection, TACE	11 (11.9)
Resection, TACE, RFA	2 (2.2)
Resection, TACE, PEIT	1 (1.1)
RFA	2 (2.2)

*Abbreviations:ECOG* Eastern Cooperative Oncology Group, *HBs-Ag* hepatitis B surface-antigen, *TACE* transarterial chemoembolization, *RFA* radiofrequency ablation, *PEIT* percutaneous ethanol injection treatment.

**Table 2:** Summary of the dosimetric parameters

**Variables**	
Normal liver volume (cm^3^)	
Median (Range)	1159.2 (488.3–1907.1)
Mean ± SD	1169.2 ± 266.7
GTV (cm^3^)	
Median (Range)	8.6 (0.6–125.3)
Mean ± SD	15.9 ± 19.3
PTV (cm^3^)	
Median (Range)	40.8 (11.9–296.9)
Mean ± SD	57.7 ± 50.0
Dose (Gy/number of fractions)	
30 / 3 – 40 / 4	31 (33.7%)
45 / 3 – 48 / 4	38 (41.3%)
60 / 3 – 60 / 4	23 (25.0%)
No. of ports	
Median (Range)	7 (5–9)
Mean liver dose (Gy)	
Median (Range)	8.3 (1.2–20.4)
Mean ± SD	8.7 ± 3.7
D_33%_* (Gy)	
Median (Range)	7.3 (0.31–28.4)
Mean ± SD	8.4 ± 5.1
D_50%_^†^ (Gy)	
Median (Range)	3.9 (0.2–16.3)
Mean ± SD	4.6 ± 3.7

*Abbreviations:SD* standard deviation, *GTV* gross tumor volume, *PTV* planning target volume.

*Dose to 33% of the normal liver.

^†^Dose to 50% of the normal liver.

### Clinical outcomes

The median follow-up period for all patients was 25.7 months (range: 1.8-55.4 months). During the observation periods, 51 patients were alive and 41 patients were deceased. The 1- and 3-year survival rates were 86.9% and 54.4% respectively; with the median survival of 53.6 months. The development of grade 2 or worse RILD did not affect the survival of patients (p = 0.099).

Radiological tumor response was evaluated in 91 patients (101 lesions) at 3 months after SBRT. Of these, 53 (51.9%) achieved complete response, 22 (21.6%) achieved partial response, and 26 (25.5%) achieved stable disease according to the modified Response Evaluation Criteria in Solid Tumors. Local control rate at 3 years was 92.1% and median time to progression was 11.1 months.

All patients received SBRT regimen as planned, and displayed no intolerable radiation-induced side effects for interruptions. Fatigue and anorexia were the most common acute toxicities; however these were mostly CTCAE grade 1.

### Univariate analysis for the risk of RILD

Of the 92 patients, 49 (53.3%) developed grade 1, 11 (11.9%) developed grade 2, and 6 (6.5%) developed ≥ grade 3 RILD after SBRT. All cases of RILD occurred within 1–3 months after SBRT. None of the patients had an abnormal liver function test during treatment.

On univariate analysis, Child-Pugh class was identified as a significant clinical parameter, while normal liver volume and normal liver volumes receiving from < 15 Gy to < 60 Gy were the significant dose–volumetric parameters. Neither the presence of HBV infection nor ECOG PS, GTV, PTV, radiation dose, fraction size, or mean dose to the normal liver contributed to the risk of developing RILD (Table 3).

**Table 3:** Univariate analyses of clinical and dose-volumetric parameters associated with the risk of radiation-induced liver disease

**Variables**	**< Grade 2 (%)**	**≥ Grade 2 (%)**	**p-value**
Age (years)	61.7 ± 8.7	59.3 ± 11.1	0.324
Gender			0.323
Male	61 (83.6)	12 (16.4)	
Female	14 (73.7)	5 (26.3)	
ECOG performance score			0.402
0-1	72 (80.9)	17 (19.1)	
2	3 (100.0)	0 (0.0)	
Child-Pugh class			0.008
A	59 (88.1)	8 (11.9)	
B	16 (64.0)	9 (36.0)	
Viral etiology			0.278
HBs-Ag (+)	58 (84.1)	11 (15.9)	
HBs-Ag (-)	17 (73.9)	6 (26.1)	
SBRT dose (Gy)	46.5 ± 9.2	43.4 ± 8.8	0.200
Fraction size (Gy)	14.0 ± 2.1	13.2 ± 1.9	0.124
Normal liver volume (cm^3^)	1199.3 ± 251.0	1036.5 ± 300.2	0.022
GTV (cm^3^)	15.9 ± 20.5	15.7 ± 12.8	0.965
PTV (cm^3^)	58.5 ± 53.5	54.4 ± 31.0	0.761
Mean liver dose (Gy)	8.6 ± 3.5	9.2 ± 4.5	0.569
D_33%_* (Gy)	8.2 ± 4.7	9.3 ± 6.8	0.435
D_50%_^†^ (Gy)	4.5 ± 3.4	5.2 ± 4.6	0.494
rV_60Gy_	1191.7 ± 249.8	1030.5 ± 300.9	0.023
rV_55Gy_	1188.8 ± 249.8	1028.8 ± 301.2	0.024
rV_50Gy_	1183.5 ± 249.0	1026.9 ± 300.9	0.027
rV_45Gy_	1167.0 ± 244.6	1015.6 ± 294.1	0.029
rV_40Gy_	1153.0 ± 244.8	999.7 ± 303.5	0.028
rV_35Gy_	1133.9 ± 243.8	977.1 ± 307.2	0.025
rV_30Gy_	1114.0 ± 243.1	959.4 ± 308.0	0.027
rV_25Gy_	1086.7 ± 241.3	936.1 ± 308.1	0.030
rV_20Gy_	1045.5 ± 239.0	900.0 ± 309.3	0.035
rV_15Gy_	976.5 ± 237.7	835.8 ± 308.0	0.040
rV_10Gy_	856.6 ± 249.9	728.9 ± 310.3	0.073
rV_5Gy_	655.5 ± 262.8	569.4 ± 295.5	0.237

*Abbreviations:ECOG* Eastern Cooperative Oncology Group, *HBs-Ag* hepatitis B surface-antigen, *SBRT* stereotactic body radiotherapy, *GTV* gross tumor volume, *PTV* planning target volume.

*Dose to 33% of the normal liver.

^†^Dose to 50% of the normal liver.

### Multivariate analysis for the risk of RILD

Multivariate analysis included only those variables with a p-value of less than 0.05, as determined in univariate analysis. To eliminate the effect of multi-colinearity among the several significant dose-volumetric parameters, only the normal liver volume receiving < 20 Gy (rV_20Gy_), which was the most significant parameter with the Hosmer-Lemeshow test, was included in multivariate analysis. Upon multivariate analysis, Child-Pugh class was the only significant parameter for predicting grade 2 or worse RILD (Table 4).

**Table 4:** Multivariate analyses of clinical and dose-volumetric parameters associated with the risk of radiation-induced liver disease

**Variables**	**p-value**	**95% CI**
Child-Pugh class	0.023	0.082–0.829
Normal liver volume	0.410	0.990–1.004
rV_20Gy_	0.792	0.994–1.008

*Abbreviation:CI* confidence interval.

## Discussion

The present study evaluated a relatively large number of patients with HCC who received high-dose irradiation (≥ 10 Gy per fraction). Upon multivariate analysis, only the Child-Pugh class was of statistical significance in predicting the risk of RILD development. By contrast, the optimal dose–volumetric parameter predictive of RILD could not be determined. The baseline liver condition is thought to be the most important factor associated with the risk of RILD after high-dose radiotherapy, even if the irradiated volume is small.

Our results showed that patients with Child-Pugh B cirrhosis more frequently developed RILD than did patients with Child-Pugh A liver function. Similar results were obtained in some previous studies of conventional radiotherapy for primary liver tumors, which concluded that baseline liver function was an important factor in predicting the occurrence of RILD [15,20]. The suggested mechanism was that a severely cirrhotic liver is less tolerable to irradiation because cirrhosis prevents the repair of radiation injury as well as the cellular proliferation, which is required to compensate for the loss of hepatic function. An association between the risk of RILD and liver function status after SBRT for HCC has been found in a limited numbers of studies. Mendez Romero et al. conducted the prospective trial of SBRT in the treatment of primary liver tumors and prescribed different dose-fraction schedules according to the tumor size and presence of cirrhosis. Acute toxicities ≥ grade 3 were observed in four patients, and one patient with HCC and Child-Pugh B liver function developed liver failure together with an infection and died. The authors therefore concluded that extreme caution is required for patients with Child-Pugh B cirrhosis due to high risk of toxicity [11]. In the series of Cardenes et al., the prescribed dose was increased to 48 Gy (16 Gy/fraction) in patients with Child-Pugh A cirrhosis without dose-limiting toxicity, however, two patients with Child-Pugh B liver function developed CTCAE grade 3 hepatic toxicities at the 42 Gy (14 Gy/fraction) level [9]. The results of these studies further support liver function as one of the most important factors in RILD, even though the irradiated volume treated with SBRT was much smaller than that targeted by 3D-CRT.

Several dose–volumetric parameters associated with RILD after 3D-CRT has been reported in the literature; however, reports evaluating acute and late hepatic toxicities after SBRT for HCC are scarce. Rusthoven et al. reported hepatotoxicity after 36–60 Gy, administered in three fractions, for the treatment of liver metastases, none of the 47 patients experienced late hepatotoxicity [12]. Lee et al. also evaluated RILD after SBRT in 68 patients with metastatic liver tumors who received a median dose of 41.8 Gy in six fractions and reported no occurrence of RILD or other grade 3–5 liver toxicity [14]. Notwithstanding these results of metastatic liver tumors, dose–volumetric parameters to predict RILD in patients with HCC who underwent SBRT were also reported. Son et al. reported that 12 patients developed grade 2 or higher hepatic toxicity after SBRT for small HCCs in 36 patients. This study focused not only on RILD but on the progression of Child-Pugh class as an endpoint for toxicity. The only significant parameter associated with the progression of Child-Pugh class (4 patients, 11%), as determined by multivariate analysis, was the total liver volume receiving a dose < 18 Gy. The authors therefore recommended that the total liver volume receiving < 18 Gy should be > 800 cm^3^, to reduce the risk of a deterioration of hepatic function [18]. Table 5 summarizes the toxicities reported in several studies of SBRT for primary and metastatic liver tumors.

**Table 5:** Comparison of toxicities of studies of SBRT for primary and metastatic liver tumors

**Study**	**Diagnosis**	**n**	**Dose prescription**	**Toxicity**
Mendez Romero [11]	HCC, Mets	25 (8, 17)	10–12.5 Gy × 3 or 5 Gy × 5 (CP B)	4 RILD ≥ grade 3, one grade 5 (HCC patient with CP B)
Tse [13]	HCC, IHC	41 (31, 10)	4–9 Gy × 6	10 (8 HCC, 2 IHC) grade 3 liver enzyme, 3 (2 HCC, 1 IHC) grade 3 hyperbilirubinemia, no grade 4–5
Rusthoven [12]	HCC, Mets	47 (2, 45)	12–20 Gy × 3	1 grade 3 soft-tissue toxicity
Lee [14]	Mets	68	4.6–10 Gy × 6	2 grade 3 liver enzyme changes (d/t DP), but no RILD
Cardenes [9]	HCC	17	12 Gy × 3 → 16 Gy × 3 (CP A) or 8 Gy × 5 (CP B)	2 grade 3 liver toxicity in patients with CP B at 42 Gy/3fx
Son [18]	HCC	36	10–13 Gy × 3	12 grade 2 or higher hepatic toxicity, 4 progression of CP
Present study	HCC	92	10–20 Gy × 3–4	11 grade 2, 6 grade 3 or higher RILD

*Abbreviations:HCC* hepatocellular carcinoma, *Mets* metastasis, *CP* Child-Pugh class, *RILD* radiation-induced liver disease, *IHC* intrahepatic cholangiocarcinoma, *DP* disease progression.

The liver function of patients with HCC is usually already compromised prior to tumor development due to the pre-existing cirrhosis after chronic liver disease, whereas this is generally not the case in patients with metastatic liver tumors. Accordingly, the QUANTEC defines a non-classic RILD and recommends that this endpoint could be appropriate in HCC patients who have poor liver function including HBV infection. They also suggest that CTCAE is most useful for scoring non-classic RILD [19]. This is the reason why we adopt non-classic RILD as the endpoint of the present study. However, we cannot neglect classic RILD which occur occasionally in patients with HCC after radiotherapy and modify the endpoint as described previously. Up to now, the definition of RILD in patients with HCC was somewhat different among the studies. Moreover, a confounder, such as the progression of cirrhosis regardless of any treatment, is an important hurdle to define RILD especially in populations with pre-existing liver dysfunction [19]. For a definite answer to RILD after SBRT, we have to overcome many obstacles to the definition of toxicity and exact meaning of laboratory findings in the follow-up examination.

In the present study, a significant dose–volumetric parameter, including mean liver dose, associated with RILD could not be identified on multivariate analysis. This is partly because SBRT is a safe modality even in patients with underlying liver disease. The median GTV (8.6 cm^3^, range 0.6–125.3 cm^3^) was smaller than that reported in the study of Son et al. (18.3 cm^3^, range 3.0–81.3 cm^3^) and the incidence of RILD ≥ grade 2 was also lower. Therefore, the incidence of RILD was not increased significantly, even though the number of patients treated with SBRT was increased. This is one of the reasons for the low statistical power of the present study. However, this is not sufficient to explain the assumption. Another possibility is that RILD may be induced by unknown causes including the production of cytokines, regardless of the irradiated normal liver volume. Hence if the mentioned assumption is true, the RILD can occur randomly after SBRT for small HCC. A dose–volumetric parameter associated with the risk of RILD could not be determined, even based on a definition of RILD as mild toxicity (grade 2). There are also no studies suggesting a normal tissue complication probability (NTCP) model to predict the risk of RILD after SBRT at doses ≥ 10 Gy per fraction. In light of the small number of cases of RILD, further studies in a larger series will be needed to construct an appropriate NTCP model.

## Conclusions

In conclusion, the Child-Pugh B cirrhosis was found to have a significantly greater susceptibility to the development of grade 2 or worse RILD after SBRT in patients with small, unresectable HCC. Additional efforts aimed at testing other models to predict the risk of RILD in a large series of HCC patients treated with SBRT are needed. Moreover, many obstacles to the definition of liver toxicity and meaning of laboratory findings should be overcome in patients with pre-existing liver dysfunction.

## Abbreviations

RILD: Radiation-induced liver disease; HCC: Hepatocellular carcinoma; SBRT: Stereotactic body radiotherapy; RFA: Radiofrequency ablation; 3D-CRT: Three-dimensional conformal radiotherapy; TACE: Transarterial chemoembolization; CT: Computed tomography; GTV: Gross tumor volume; ITV: Internal target volume; PTV: Planning target volume; OBI: On-board imager; CTCAE: The Common terminology criteria for adverse event; QUANTEC: The Quantitative analyses of normal tissue effects in the clinic; HBV: Hepatitis B virus; ECOG PS: Eastern cooperative oncology group performance score; NTCP: Normal tissue complication probability.

## Competing interests

The authors declare that they have no competing interests.

## Authors’ contributions

Each author had participated sufficiently in the work to take public responsibility for appropriate portions of the content. JJ and SMY participated in research design, coded the patient database, conducted the analysis and wrote the manuscript draft and revised the manuscript. SYK, BC, and SSK designed the project, analyzed the data and revised the manuscript. SL and JHK contributed to conception and study design. JP and SYS helped with the database and data analysis. SDA and EKC provided additional guidance and support for this research. All authors read and approved the final manuscript.
